# Physicochemical Compatibility Investigation of Mesalazine and Folic Acid Using Chromatographic and Thermoanalytical Techniques

**DOI:** 10.3390/ph13080187

**Published:** 2020-08-08

**Authors:** Mario-Livio Jeličić, Edvin Brusač, Daniela Amidžić Klarić, Biljana Nigović, Sabina Keser, Ana Mornar

**Affiliations:** Faculty of Pharmacy and Biochemistry, University of Zagreb, A. Kovačića 1, 10000 Zagreb, Croatia; mljelicic@pharma.hr (M.-L.J.); ebrusac@pharma.hr (E.B.); damidzic@pharma.hr (D.A.K.); bnigovic@pharma.hr (B.N.); skeser@pharma.hr (S.K.)

**Keywords:** mesalazine, folic acid, inflammatory bowel disease, drug compatibility, fixed-dose combination

## Abstract

Inflammatory bowel disease is a common name for Crohn’s disease and ulcerative colitis. These inflammatory states cause damage in the sidewalls of the gastrointestinal tract, resulting in malabsorption of food and vitamins. Folic acid (Vitamin B9) is often associated with inflammatory bowel diseases since reduced overall folate concentration in the human body may lead to the development of colorectal cancer and megaloblastic anaemia. However, its deficiency is easily compensated by taking an additional folic acid pill during regular therapy. At the moment, there are no studies that have examined the compatibility of folic acid with 5-aminosalicylate drugs used in the treatment of inflammatory bowel diseases. In this work, differential scanning calorimetry, forced degradation studies, isothermal stress testing and dissolution stability testing were used to determine the stability of folic acid and one of the most commonly used 5-aminosalicylates, mesalazine, when present in the same solution or blend. To monitor the assay of folic acid, mesalazine and nine of its related impurities, a single HPLC method was developed. Results of compatibility studies showed that no physicochemical interaction between mesalazine and folic acid occurs when combined, opening the path to the development of new formulations, such as a mesalazine/folic acid fixed-dose combination.

## 1. Introduction

Incompatibility of two or more drugs is defined as a change in a product which leads to unacceptable stability, safety and efficacy, and comes in a form of physical, chemical or therapeutic incompatibility. Physical incompatibility can emerge when two or more substances are combined, forming an unwanted product, which can be observed as a change in colour, taste, odour or morphology. Chemical incompatibility changes the effectiveness of a drug by forming inactive or toxic products as a result of chemical change in the drug itself. Therapeutic incompatibility of two drugs can emerge when two or more drugs are administered simultaneously, affecting each other’s pharmacokinetics by changing the absorption, distribution, metabolism and excretion, or by having an impact on pharmacodynamics, subsequently resulting in synergistic or antagonistic effects [1,2]. The process of combining drugs has to be approached with care in order to prevent the occurrence of drug incompatibility, hence increasing the therapy effectiveness and safety.

Mesalazine (MSZ) is one of the most prescribed 5-aminosalicylate drugs in the treatment of inflammatory bowel diseases (IBD). It is an anti-inflammatory drug that acts locally on the site of inflammation in the gastrointestinal tract. MSZ has been used for the treatment of IBD for over four decades and was successful in the induction and maintenance of remission [3]. On the other hand, folic acid (FA) is often taken simultaneously with 5-aminosalicylates in order to reduce the risk of birth defects in pregnant women, colorectal cancer and megaloblastic anaemia development in patients with IBD [4,5,6,7,8]. As IBD damages parts of the gastrointestinal tract, the overall surface for absorption of FA is reduced, resulting in its malabsorption. Therefore, IBD patients are often prescribed elevated doses of FA (1–5 mg/day) compared to non-IBD patients (0.4 mg/day) [9,10]. Therapies in which multiple drugs have to be taken simultaneously, as in IBD, often raise the question of medication adherence, as it is important to take FA regularly.

Currently, there is no reported study on the physicochemical compatibility of MSZ and FA. Drug-drug incompatibility testing is approached using various studies to gather as much information as possible about the potential drug-drug interaction. Differential scanning calorimetry (DSC), a thermoanalytical technique, is commonly used in the screening of drug-excipient blends, solely based on shift or disappearance/occurrence of endothermic or exothermic peaks, however, the same principle can be applied to drug-drug combinations [11,12,13]. Forced degradation studies are applied to induce the formation of representative degradation products as much as possible in various conditions in order to develop a stability-indicating method for further studies. This approach is useful for drug-drug compatibility tests, as it allows one to obtain insight into the formation of new degradation products by comparing drugs stressed in combination vs. separately [14,15]. Isothermal stress testing in combination with the developed stability-indicating method is used for the monitoring of drug-drug and drug-excipient compatibility by storing the blends in conditions of elevated temperatures and controlled humidity for longer periods to monitor physicochemical interactions [16]. The dissolution method represents the approach in which the drug is dissolved in different biorelevant media in order to monitor drug release and drug stability. This approach was successfully applied in drug-drug and drug-excipients compatibility studies [17]. Considering the results obtained by all these methods, a wider picture can be obtained and conclusions can be made regarding the compatibility of the two drugs.

**Figure 1:**
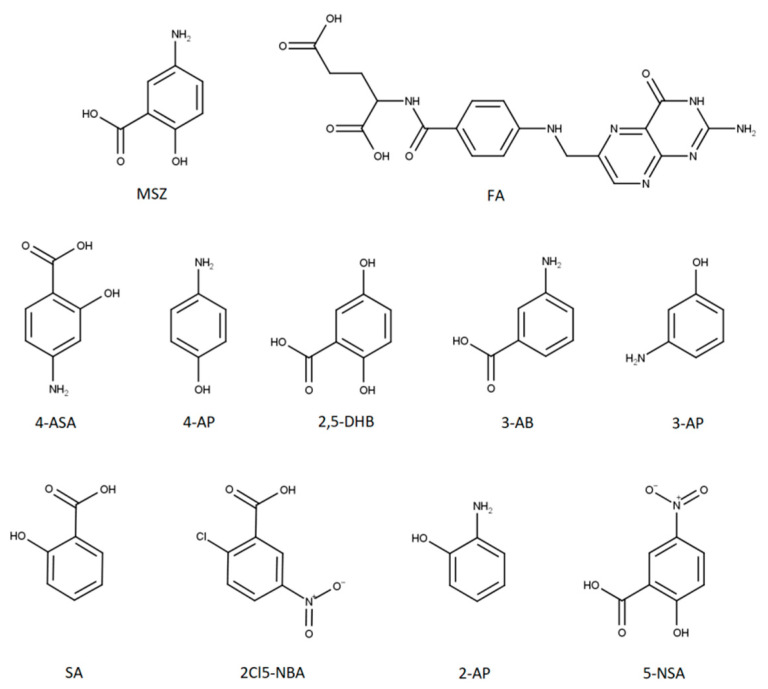
Structures of mesalazine (MSZ), folic acid (FA) and MSZ-related impurities.

The aim of this work was to examine the possible interactions between MSZ and FA when present together in a homogenous blend or solution to open the path to the development of MSZ/FA fixed-dose combinations. MSZ and FA blends contained the amount of each drug in a ratio which would ensure the maintenance of the remission state and supplement the malabsorbed FA during the therapy of IBD. Preliminary thermoanalytical measurements using DSC were conducted followed by forced degradation studies and isothermal stress testing. In the end, dissolution tests using simulated gastric and intestinal fluids were carried out to examine the possible interactions and their stability in such media. In order to monitor the degradation rate of MSZ and FA, an analytical method was developed for the detection and quantification of both active pharmaceutical ingredients (APIs) as well as nine MSZ-related impurities: 4-aminosalicylic acid (4-ASA), 4-aminophenol (4-AP), 2,5-dihydroxybenzoic acid (2,5-DHB), 3-aminobenzoic acid (3-AB), 3-aminophenol (3-AP), salicylic acid (SA), 2-chloro-5-nitrobenzoic acid (2Cl5-NBA), 2-aminophenol (2-AP) and 5-nitrosalicylic acid (5-NSA) (Figure 1).

## 2. Results and Discussion

### 2.1. Differential Scanning Calorimetry

**Figure 2:**
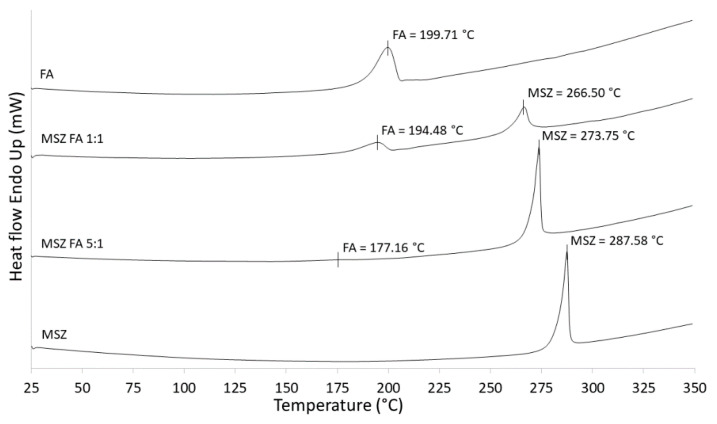
Differential scanning calorimetry (DSC) curves of MSZ, FA and the prepared blends.

The results of DSC scans of MSZ and FA as well as MSZ/FA standard blends in a ratio of 1:1 and 5:1 are shown in Figure 2. The endothermic peak of the MSZ standard occurs at 287.58 °C, which matches the MSZ melting point in the literature [18]. On the other hand, in the literature [19,20], FA only has a reported decomposition temperature at approximately 250 °C, at which it begins to darken/char and a significant weight loss is observed. However, it does not have a reported melting temperature.

In our measurements, a clear endothermic peak at 199.71 °C is visible and it is possibly due to the melting. In DSC, when screening MSZ/FA 5:1 and 1:1 blends, a shift in the MSZ peak to lower temperatures was observed, resulting in temperatures of 273.75 and 266.50 °C, respectively. In the case of FA, a significant shift in peak was observed (177.16 °C) in the 5:1 blend, while in the 1:1 blend, the temperature was less shifted (194.48 °C). In all measurements, in spite of the obvious shift in temperature, peak shapes remained similar without noticeable broadening or disappearance. If the obtained DSC curve of a blend shows a shift in temperature to a lower or higher value (more than 5 °C) with absence of existing or occurrence of new endothermic or exothermic peaks when compared to the same compound alone, then there is a possibility that incompatibility might occur. Considering that the observed shifts in temperatures were higher than 5.23 °C, there was a doubt that MSZ and FA are incompatible; however, conclusions cannot be made solely on DSC measurements and there was a need for the use of other complementary methods [21,22].

### 2.2. HPLC Method Development

MSZ is a hydrophilic, polar molecule with a poor retention time on reversed-phase columns. Moreover, its impurities are structurally very similar and also polar; therefore, the goal was to develop a single method to separate MSZ, nine of its related impurities and FA up to an acceptable resolution. Column performance plays an important role in peak separation, especially when close-eluting peaks are expected. The column with smaller particles (2.7 µm), such as the Cortecs Phenyl column, was shown to be superior over standard columns (5 µm) due to narrower and higher eluting peaks that increased the separation efficiency.

Different compositions of mobile phases were tested in order to achieve the best chromatographic performance. Methanol, being a weaker eluent than acetonitrile, has shown to be a better choice since the components are more retained by the stationary phase of the column. Examining the various mobile phase additives, the best separation and peak performance was obtained using the ammonium acetate buffer adjusted to pH 5.0 ± 0.1 with glacial acetic acid.

**Figure 3:**
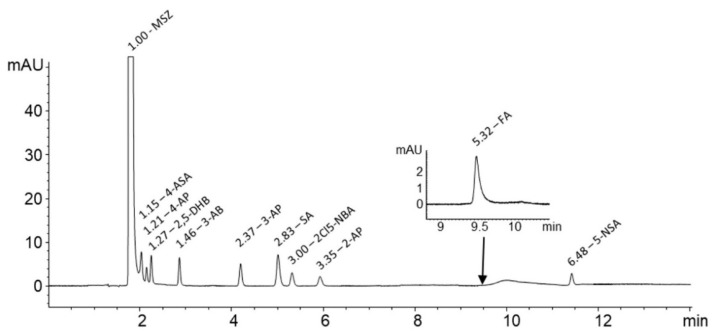
Chromatogram of MSZ and its related impurities (100% concentration level) at 240 nm. Insert shows FA peak at 285 nm. Hump at the 10th minute is due to the gradient.

The temperature was shown to have a strong impact on column performance. With the increment of temperature from 25.0 up to 50.0 °C, the peak characteristics were greatly improved. At 50.0 ± 0.1 °C column temperature, the peaks were noticeably narrower without shortening their retention time. Furthermore, different flow rates were tested in a range of from 0.4 mL/min up to 1.0 mL/min. No significant improvement in performance was observed with lowering the flow rate, therefore, it was kept constant at 1 mL/min. MSZ and FA were detected at 240 nm and 285 nm, while MSZ-related impurities were detected at 220 nm. The chromatogram obtained with the final chromatographic conditions showing all peaks with accompanied relative retention times (RRT) in relation to MSZ is shown in Figure 3.

### 2.3. Method Validation and Application

The method was validated as per the International Council for Harmonisation of Technical Requirements for Pharmaceuticals for Human Use (ICH) guideline Q2 (R1) for selectivity, linearity, precision, accuracy, limit of detection, limit of quantification and robustness [23].

The selectivity of the method was determined by the visual examination of chromatograms obtained with the analysis of excipient solutions held in the same conditions as in each conducted degradation. No peaks were observed at the times of the elution of standards. Resolution between all peaks was higher than 1.5 and the peak purity of each peak was calculated where all values were higher than 998.

The linearity of the method was determined on five concentration levels in the range of from 25% to 120% of the maximum allowed concentration according to British Pharmacopoeia (BP). Linearity ranges, calibration curve equations and accompanying correlation coefficients are shown in Table 1. High values of correlation coefficients (*R* ≥ 0.998) imply that satisfactory linearity was obtained in the given range.

Method precision was determined as inter- and intra-day precision at a level of 100%. Intra-day precision was studied with the analysis of six separately prepared samples, where all samples were analysed during the same day. Inter-day precision was carried out with the preparation and analysis of three samples each day for three consecutive days. Obtained results are expressed as relative standard deviation (% RSD) and are shown in Table 2. As can be seen from provided data, all RSD values never exceeded 3.80% and 3.83% for intra- and inter-day precision, respectively, which implies that the method is precise.

Accuracy of the method was expressed as recovery and was tested in triplicates on three concentration levels (25%, 100% and 120%) in order to cover the whole range of the calibration curve. Results are shown in Table 2 as a percentage of recovery with an accompanied RSD value. RSD values never exceeded 3.13%.

The method was shown to be robust regarding the retention times (RSD < 5%) on changes in flow rate (1.00 ± 0.02 mL/min), column temperature (50.0 ± 1.0 °C), pH of the buffered mobile phase (5.0 ± 0.2) and changes in gradient (±1% of methanol). The resolution between the close-eluting peaks remained above 1.5 with all changes applied.

The MSZ standard and tablets used in further studies were analysed to determine the amount of impurities using the developed method. In the MSZ standard, 2,5-DHB impurity was found, albeit below the limit of quantification. Analysis of the MSZ tablet showed that 2,5-DHB and SA impurities were present. Moreover, 2,5-DHB was present in a concentration of 0.08 µg/mL, while SA was below the limit of quantification. The concentrations of impurities found in the standard and tablet samples are below or near the limit of quantification and will not impact the results of forced degradation studies.

### 2.4. Forced Degradation Study

The goal was to achieve optimal degradation from 5% up to a maximum of 20%. Degradations higher than 20% were avoided due to the fact that formed degradation products could also degrade in the same conditions, giving additional peaks that could mislead the course of method development [24]. Furthermore, extremely harsh conditions were avoided to eliminate the rapid degradation of the examined API to eliminate the creation of non-representative degradation products. Each API was subjected to degradation separately in order to examine the best degradation conditions. The time needed for the first API to degrade (in between 5% and 20%) was used as the optimal time for degradation, even if the other one did not degrade significantly in that period. The same principle was applied for experiments with standard and tablet blends.

**Figure 4:**
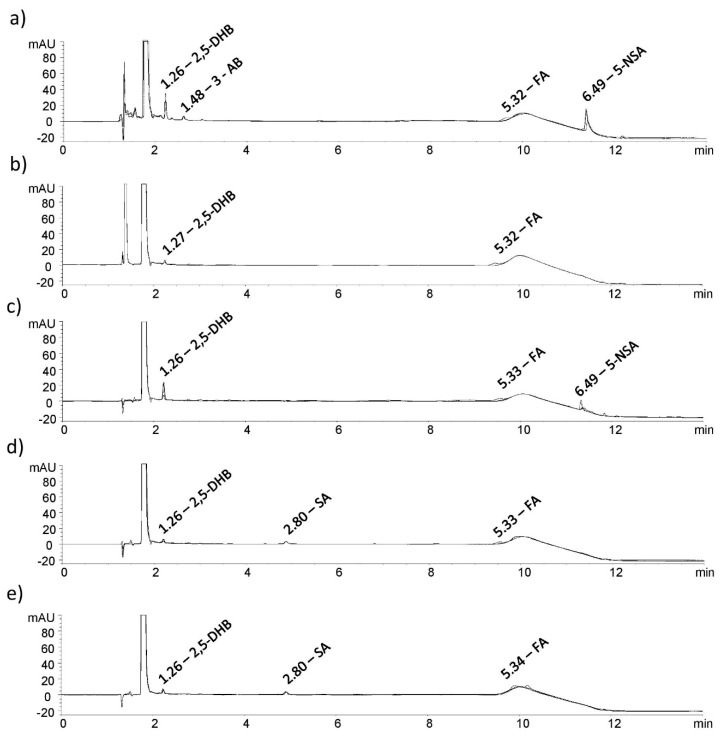
Overlapped chromatograms of stressed MSZ, FA and their combinations in: (**a**) alkali, (**b**) oxidative, (**c**) photo (solution), (**d**) photo (solid) and (**e**) thermal (solution) conditions.

Chromatograms obtained from the analysis of the samples that were subjected to the stress study were examined in order to determine the presence of new major unknown peaks (Figure 4).

In this manner the impact of MSZ on FA and vice versa could be determined as a change in the degradation rate and through the appearance of new unidentified peaks in the mixed samples. The optimal degradation times and percentage of sample degradation are presented in Table 3.

#### 2.4.1. Alkali Degradation

The prepared samples were kept in alkali conditions (0.1 M NaOH) for one hour and it was observed that MSZ was unstable in such conditions. A drop in the MSZ assay of 9.4% and in the FA assay of 2.1% was measured for alkali degradation of each API separately. The standard blend and tablet blend, also kept in the same conditions, showed similar degradations. Reductions in the MSZ assays of 11.6% and 10.4% and FA assays of 1.4% and 1.6% were observed. Considering the concentration of the FA in solution (2 µg/mL) and the related assay drops, it can be assumed that FA is stable in alkali conditions alone and in the presence of MSZ. The overlapped chromatograms showing MSZ impurities caused by alkali degradation are shown in Figure 4a. Three degradant products emerged: the peak at RRT of 1.26 corresponds to 2,5-DHB, the peak at 1.48 corresponds to 3-AB and that at 6.49 RRT corresponds to 5-NSA. Furthermore, by visually examining the obtained chromatograms, no new major peaks evolved as a consequence of FA being mixed with MSZ.

#### 2.4.2. Acid Degradation

Keeping the standards of MSZ and FA separately in acidic conditions (0.1 M HCl) the assay of FA in the solution was reduced by 10.6% after 30 min of reaction time. MSZ showed greater stability during the same period with only 0.8% of assay reduction. The standard blend and tablet blend were also kept under the same conditions for 30 min; the FA assay was reduced by 8.7% and 6.0% while MSZ assay by 0.5% and 0.1%, respectively. Comparing the given results, FA was shown to be slightly more stable in the blend than when separated. This could be due to the fact that when combined, MSZ is present in a far greater concentration, reducing the chance of FA to be hydrolysed. Considering the low degradation percentage of MSZ, no new major peaks were observed.

#### 2.4.3. Oxidative Stress

Samples were exposed to oxidative stress using a 3% hydrogen peroxide solution. The reaction time of 1 h was required for the MSZ assay to drop by 9.1%. The FA sample showed to be stable for that period with only a 0.7% assay reduction. The standard blend showed almost identical behaviour with a 9.8% reduction in the MSZ assay and a 0.7% reduction in the FA assay. The MSZ and FA assays in the tablet blend were similarly reduced, with a decrease of 10.3% and 1.0%, respectively. Overlapped chromatograms of the stressed samples are shown in Figure 4b. The only peak that emerged after the reactions was that at 1.27 RRT which corresponds to 2,5-DHB.

#### 2.4.4. Photodegradation

Solutions and solids of the MSZ and FA standards, their blend and the tablet blend were continuously exposed to indirect daylight. After seven days of maintaining exposure of the solid samples, degradations in the range of 6.4% to 4.2% for MSZ were measured, while for the FA assay, reductions were in range of 6.5% to 2.9%. Considering the conditions to which the samples were exposed and the observed degradations, it can be assumed that MSZ and the FA solid samples are relatively stable. When examining the overlapped chromatograms, two peaks emerged. RRT of 1.26 and 2.80 corresponds to 2,5-DHB and SA, respectively (Figure 4c). Considering the results obtained above, the prepared MSZ and FA formulations should be kept protected from light to reduce photolytic degradation over a longer period.

After four hours of exposing the standard solutions to light, a drop in the MSZ assay of 2.9% was observed. On the other hand, as expected, the FA solution was shown to be very sensitive to light, displaying a reduction of 18.3% during the same period [25]. By keeping the standard blend solution exposed to the light, interesting results were obtained. After exposing the solution for 4 h, FA concentration changed insignificantly. However, by exposing the solution for an additional 8 h to the light, FA concentration dropped by only 4.6%, while MSZ degraded by 13.4%. It can be concluded that FA is shielded by MSZ molecules, notably reducing the chance of FA exposure to UV light. Similar results were obtained with the solution of the prepared tablet blend as with the standard blend solution. A reduction in the assay of 11.1% and 2.3% for MSZ and FA, respectively, shows that MSZ presence in greater concentration than FA has a major impact on FA stability in solutions. When examining the overlapped chromatograms, two peaks emerged. RRT of 1.26 and 6.49 corresponds to 2,5-DHB and 5-NSA, respectively (Figure 4d).

#### 2.4.5. Thermal Degradation

As in the photolytic stress study, both the solution and solid samples were subjected to thermal degradation. The MSZ solution appeared to degrade at higher temperatures during the period of 18 h. Assay reductions of 15.4%, 11.1% and 13.0% were measured for the MSZ standard, the standard blend and the tablet blend, respectively. On the other hand, FA is shown to be stable at a set temperature with maximum degradation, as in the case of the tablet blend (2.8%). Inspecting the obtained chromatograms, only two peaks emerged as a result of MSZ degradation, at RRT of 1.26 and 2.80, corresponding to 2,5-DHB and SA, respectively (Figure 4e).

Solid samples were kept in the same conditions, and assay reductions of 9.0%, 7.4% and 8.6% were measured for the MSZ standard, the standard blend and the tablet blend, respectively. On the other hand, FA showed no degradation whatsoever. No significant degradation peaks were observed in this study.

### 2.5. Isothermal Stress Testing

Isothermal stress testing is a common technique used in compatibility studies which involves keeping the samples at higher temperatures with or without moisture in order to induce the possible interactions between the components of the sample [26]. It is followed by HPLC measurements to determine the assay of the components after their storage, or other techniques to observe any other changes. Any noticeable drops in the assay of any component may indicate that there is possible interaction/incompatibility. Results of the conducted stress testing on both MSZ and FA standards, their combination as well as on each tablet and the prepared tablet blends are presented in Table 4.

All samples were visually inspected before and after the storage period of 4 weeks, and no changes in the appearance of the samples were observed. Recoveries of all samples are in the range of 98.80% to 103.56% with a maximum RSD of 2.23%. These results indicate that there was no chemical reaction between MSZ and FA. Any notable incompatibility between MSZ and FA could result in a significant drop in the FA assay, considering that MSZ is present in s far greater share in the prepared blends.

### 2.6. In Vitro Dissolution Testing

**Figure 5:**
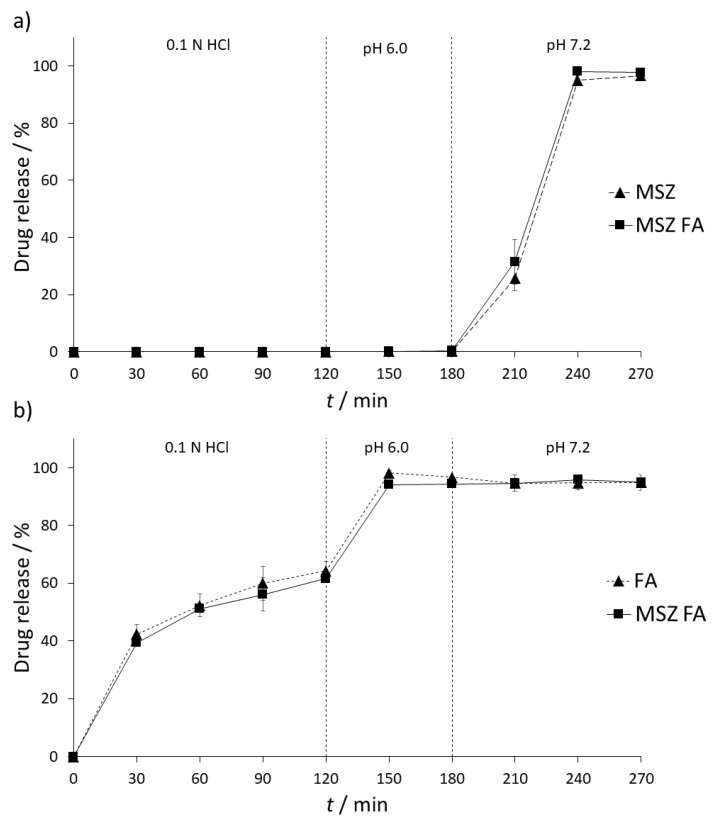
Drug release profiles of: (**a**) MSZ and (**b**) FA alone and in combination in different dissolution media (*n* = 3).

Dissolution studies were reported to be used for the determination of the stability of two drugs when present in media that simulates the gastric and intestinal fluids [27,28]. The stability of MSZ and FA tablets and their combination was examined in United States Pharmacopoeia (USP)-simulated gastric and intestinal fluids (SGF, 0.1 N HCl and SIF, pH 6.0 and 7.2). Due to local acting of MSZ and the fact that it can be easily absorbed before reaching inflamed parts of the gastrointestinal tract, it comes in the form of a gastro-resistant tablet; therefore, it is not expected to see any interaction between MSZ and FA in gastric conditions. Results of dissolution testing of each tablet and their combination are shown in Figure 5.

As expected, there was no release of MSZ from the formulation in both SGF and SIF (pH 6.0) dissolution media. Changing the SIF pH to 7.2 resulted in a gradual release of MSZ. Over the period of one and a half hours, up to 96.69% ± 1.49% of MSZ was released from the formulation (Figure 5a). On the other hand, a slow release of FA was detected in SGF, with a maximum release of 64.29% ± 3.18% over a period of two hours. In SIF (pH 6.0), complete release of FA was observed (96.75% ± 0.08%). FA remained stable over the whole period of SIF (7.2) conditions (96.76% ± 0.06%) (Figure 5b).

Possible interferences between MSZ and FA were tested by placing both tablets in the same dissolution vessel. No interaction between MSZ and FA was expected during the SGF and SIF (6.0) stage since MSZ is not released at this stage; however, since FA is released in both stages, possible interaction between FA and the MSZ tablet coating could occur, resulting in premature release of MSZ from the formulation. In both stages, FA did not have any impact on the release of MSZ and the amount of released FA was similar to that when not combined (94.35% ± 0.31%). Furthermore, no interaction between MSZ and FA was observed upon increasing the dissolution media pH to 7.2, which resulted in the release and dissolution of MSZ. Upon the end of experiment, the recovered FA assay was 94.97% ± 0.89%, implying that FA remained stable in the dissolution media in the presence of MSZ and that no chemical or physical interaction occurred.

In order to confirm the similarity of the obtained dissolution curves, difference (*f*_1_) and similarity factors (*f*_2_) were calculated. Difference factor is a measure of relative error between two curves and is expressed as a difference percentage, with values between 0 and 15% as acceptance criteria. The difference factor for MSZ-MSZ/FA dissolution profiles is 4.51%, while for FA-MSZ/FA profiles it is 3.16%. These values indicate that there is no significant difference between the two curves. On the other hand, similarity factor (*f*_2_) is a measure of similarity of two curves; values from 100% to 50% are considered acceptable where curves are identical if *f*_2_ value is 100%. In the case of MSZ-MSZ/FA and FA-MSZ/FA, *f*_2_ values were 82.09% and 77.01%, respectively, implying that the two obtained dissolution profiles are similar.

## 3. Materials and Methods

### 3.1. Reagents and Chemicals

MSZ and FA certified reference standards were provided by European Pharmacopoeia and MSZ-related impurities, namely, 4-ASA (99%), 4-AP (>98.0%), 2,5-DHB (>99.0%), 3-AB (>99.0%), 3-AP (>98.5%), SA (≥99.0%), 2Cl5-NBA (97.0%), 2-AP (>98.0%), and 5-NSA (>98%), were provided by Sigma Aldrich (St. Louis, MO, USA). The following chemicals were used for pH adjustment, the mobile phase and buffer creation: hydrochloric acid 37% (Carlo Erba, Val-de-Reuil, France), sodium hydroxide pellets ≥98.0% (Sigma Aldrich), hydrogen peroxide 30% (T.T.T. d.o.o., Sveta Nedelja, Croatia), glacial acetic acid (Panreac Química S.L.U., Barcelona, Spain), potassium phosphate and dipotassium phosphate (Kemika, Zagreb, Croatia) and acetonitrile and methanol HPLC-grade solvents by J.T. Baker (Avantor, Deventer, Netherlands). For selectivity tests and method validation, the following excipients were used: hydroxypropyl methylcellulose Methocel K100M Premium CR (Colorcon, Harleysville, PA, USA), magnesium stearate (Acros Organics, Princeton, NJ, USA), lactose monohydrate, stearic acid, wheat, rice and corn starch (Kemig, Zagreb, Croatia).

For experiments which required finished product, Salofalk^®^ 500 mg gastro-resistant tablets, (Dr. Falk Pharma GmbH, Freiburg, Germany) and folacin 5 mg tablets (Jadran-Galenski Laboratorij d.d., Rijeka, Croatia) were used.

### 3.2. Preparation of Blends of Standards and Tablets

The blends of standards in the proposed ratio was prepared by carefully weighing and mixing 1000 mg of the MSZ standard with 2 mg of the FA standard. Powders were thoroughly mixed over a period of 20 min to achieve complete homogenisation. Five Salofalk^®^ tablets containing 500 mg of MSZ were weighed and ground to a fine powder. The amount of powder equal to 2000 mg of MSZ was carefully weighed and mixed with the folacin powder equal to 4 mg of FA in order to achieve the desired ratio. Tablet powders were thoroughly mixed over a period of 20 min in order to achieve complete homogenisation.

### 3.3. Preparation of Standard and Working Solutions

All MSZ impurity standards used in the development of the HPLC method were carefully weighed and dissolved in water:methanol (90:10 *v*/*v*), and sonicated for 10 min in an ultrasonic bath (Elmsonic XtraTT, Biosan, Riga, Latvia) maintained at 40.0 °C. The final working solution containing 1000 µg/mL MSZ, 2 µg/mL FA, 0.02 µg /mL 4-AP and 2-AP, 0.10 µg/mL 3-AP, 5-NSA and 3-AB, 0.15 µg/mL 2,5-DHB, 4-ASA and 2Cl5-NBA, and 0.30 µg/mL SA represents the maximum allowed concentration of impurities to be present in the formulation as per the British Pharmacopoeia (BP) monograph for the MSZ finished product [29]. All samples were prepared with the addition of a mixture of commonly used excipients to the standard samples in a ratio that is expected in commercially available products in order to replicate the matrix of the real sample.

### 3.4. Chromatographic Conditions

The Agilent 1100 series HPLC system (Agilent Technologies, Waldbronn, Germany) coupled with a diode array detector with Chemstation for data processing was used for all chromatographic analyses. A Cortecs Phenyl column (150 × 4.6 mm, particle size 2.7 µm) with a suitable guard column, both obtained by Waters (Milford, MA, USA), was used for chromatographic separation. The column was maintained at 50.0 ± 0.1 °C during the analysis, while the flow rate was kept constant at 1.0 mL/min. Mobile phase A consisted of 5.0 mM ammonium acetate buffer (adjusted to pH 5.0 ± 0.1 with glacial acetic acid using the FiveEasy pH meter by Mettler Toledo, Columbus, OH, USA), while methanol was used as mobile phase B. Gradient elution was applied as follows: 0–7 min, isocratic 5% B; 7–9 min, linear gradient 5–20% B; 9–16 min, isocratic 20% B; 16–20 min, isocratic 5% B. The analysis time was 16 min and the total run time was 20 min to allow re-equilibration of the stationary phase for the following analysis. A sample volume of 5.0 µL was injected into the chromatographic system. MSZ and its related impurities were detected at 240 nm and 220 nm, respectively, whilst FA was monitored at 285 nm.

### 3.5. Differential Scanning Calorimetry

DSC curves were obtained using the Perkin-Elmer Diamond differential scanning calorimeter (Perkin Elmer, Inc., Waltham, MA, USA), calibrated with indium (99.98% purity; melting point 156.61 °C and fusion enthalpy of 28.71 J/g). Samples were carefully weighed (3–5 mg) directly into 50 µL aluminium pans and closed with a pierced aluminium lid. Measurements were carried under the atmosphere of pure nitrogen in a flow rate of 25 mL/min at a heating rate of 10 °C/min. The heating temperature for all samples was in a range from 25 to 350 °C. All samples were measured in duplicates.

### 3.6. Forced Degradation Study Conditions

All stress studies were conducted on samples containing 1000 µg/mL of MSZ and 2 µg/mL of FA when stressed separately or in combination. Samples were kept in stress conditions until optimal degradation of 5–20%. For each stress study, control samples were prepared and kept in the dark at room temperature for the same period. All samples, after the reaction time, were prepared and analysed using the chromatographic procedure described in Section 3.4.

Acid, alkali and oxidative degradations were conducted on solutions prepared by carefully weighing the standards and the prepared blends and dissolving them in water:methanol (90:10 *v*/*v*). Samples were sonicated for 15 min to achieve complete dissolution, and aliquot of 1 M NaOH, 1 M HCl or 30% H_2_O_2_ was added to each vial depending on the conducted stress study. The final conditions were: 0.1 M NaOH, 0.1 M HCl and 3% H_2_O_2_. The vials were sealed and stored in the dark at room temperature during the reaction time. The reaction time of 30 min was needed for optimal degradation in acidic conditions and 60 min in alkaline and oxidative conditions.

For photodegradation and heat degradations, the standards and the prepared blends were carefully weighed directly into a transparent HPLC vial separately and dissolved in 1 mL of water:methanol (90:10 *v*/*v*). Powders of the MSZ and FA standards, as well as the standard and tablet blend, were spread in a thin layer on a Petri dish. The prepared solutions and solid samples were exposed to indirect daylight. The solid samples were kept exposed to indirect daylight for 7 days, while the solutions were exposed for 4 h in the case of the FA solution and 12 h in the case of solutions containing MSZ. The effect of thermal stress was determined by placing the prepared solutions and solids in an ES-20/60 Orbital Shaker-Incubator by Biosan (Riga, Latvia) and thermostated at 70 °C. The solutions were heated for 18 h, while the solid samples were heated up to 7 days.

### 3.7. Isothermal Stress Testing

The MSZ and FA standards and their blends as well as the tablet powders and their blends were visually inspected and placed in the ES-20/60 Orbital Shaker-Incubator and thermostated at 50 °C for 4 weeks. Samples were examined to identify any visual changes and dissolved in water:methanol (90:10 *v*/*v*) to achieve a concentration of 1000 µg/mL MSZ and 2 µg/mL FA. Samples were filtered and injected into the HPLC system. Control samples were prepared and stored in the dark at room temperature for the same period.

### 3.8. In Vitro Dissolution Studies

Dissolution of MSZ, FA and their combination was examined using the USP method for dissolution of MSZ delayed-release tablets [30]. Testing was performed on USP 2 apparatus LDLT-A10 (Labtron Equipment Ltd., Fleet, UK) in three different dissolution media: simulated gastric fluid (SGF, 0.1 N HCl) and phosphate buffer solutions of pH 6.0 (stage 1) and 7.2 (stage 2), both also known as simulated intestinal fluids (SIF). The dissolution media volume was kept constant at 500 mL, while the paddle rotation speed was 100 rpm for acid and buffer stage 1 and 50 for buffer stage 2. Samples were taken every 30 min from the dissolution vessel, filtered and injected into the HPLC system for assay determination. The drawn sample volume was replenished with an equal amount of fresh dissolution media. All measurements were conducted in triplicate.

## 4. Conclusions

Based on the obtained results, it can be considered that FA and MSZ have no chemical nor physical interaction. Although DSC measurements showed possible incompatibility, forced degradation studies and isothermal stress testing showed that there is no significant interaction when the drugs are combined as a blend or in a solution. Moreover, if MSZ and FA had any chemical interaction, it would be observed unambiguously, as the FA assay would be significantly reduced since it is 500 times less present in the sample. This research can open the path to possible development of an MSZ and FA fixed-dose combination, considering the importance of FA compensation since medication adherence is one of the problems in polypharmacy therapy of chronic diseases.

## Figures and Tables

**Table 1:** Method calibration data.

Analyte ^1^	RRT ^2^	Linearity Range (µg/mL)	Equation	*R* ^3^	Limit of Detection (µg/mL)	Limit of Quantification (µg/mL)
**Active Pharmaceutical Ingredients**						
MSZ	1.00	500–1500	*y* = 2.3629*x* + 273.1	0.999	-	-
FA	5.32	1.00–3.00	*y* = 22.987*x* − 0.845	0.999	0.30	1.00
**Impurities**						
4-ASA	1.15	0.375–1.875	*y* = 10.406*x* + 0.005	0.998	0.02	0.07
4-AP	1.21	0.25–1.25	*y* = 11.882*x* + 0.145	0.999	0.03	0.09
2,5-DHB	1.27	0.375–1.875	*y* = 17.735*x* + 2.553	0.999	0.02	0.07
3-AB	1.46	0.25–1.25	*y* = 36.573*x* + 0.129	0.999	0.01	0.05
3-AP	2.37	0.25–1.25	*y* = 25.545*x* + 0.389	0.999	0.02	0.06
SA	2.83	0.75–3.75	*y* = 13.102*x* + 0.041	0.999	0.04	0.13
2Cl5-NBA	3.00	0.375–1.875	*y* = 13.185*x* + 0.101	0.999	0.04	0.15
2-AP	3.35	0.25–1.25	*y* = 14.757*x* + 0.439	0.999	0.04	0.14
5-NSA	6.48	0.25–1.25	*y =* 15.720*x* − 0.571	0.999	0.03	0.11

^1^ Analyte: mesalazine (MSZ), folic acid (FA), 4-aminosalicylic acid (4-ASA), 4-aminophenol (4-AP), 2,5-dihydroxybenzoic acid (2,5-DHB), 3-aminobenzoic acid (3-AB), 3-aminophenol (3-AP), salicylic acid (SA), 2-chloro-5-nitrobenzoic acid (2Cl5-NBA), 2-aminophenol (2-AP) and 5-nitrosalicylic acid (5-NSA); ^2^ relative retention time; ^3^ correlation coefficient.

**Table 2:** Precision and accuracy data.

Analyte ^1^	Precision as Relative Standard Deviation (RSD) (%)		Accuracy as Recovery ± RSD (%)		
	Intra-Day Precision (*n* = 6)	Inter-Day Precision (*n* = 9)	Low (*n* = 3)	Medium (*n* = 3)	High (*n* = 3)
**Active Pharmaceutical Ingredients**					
MSZ	0.77	0.78	97.88 ± 0.99	99.23 ± 0.61	99.44 ± 0.05
FA	1.31	1.56	103.60 ± 0.83	99.24 ± 2.11	100.23 ± 0.59
**Impurities**					
4-ASA	0.65	1.29	102.02 ± 1.02	99.36 ± 2.05	99.90 ± 0.66
4-AP	1.49	1.71	101.02 ± 0.47	97.61 ± 1.58	99.44 ± 0.82
2,5-DHB	1.56	1.64	100.27 ± 0.54	99.36 ± 1.18	99.90 ± 1.04
3-AB	1.03	1.05	100.02 ± 0.34	99.14 ± 0.65	98.97 ± 1.50
3-AP	1.14	1.64	102.03 ± 1.38	98.48 ± 1.96	98.60 ± 1.99
SA	0.89	0.99	98.83 ± 1.67	100.50 ± 0.67	99.18 ± 0.87
2Cl5-NBA	1.51	2.57	100.15 ± 2.11	100.10 ± 3.09	98.72 ± 1.36
2-AP	3.80	3.83	101.08 ± 3.13	98.01 ± 2.99	101.65 ± 2.94
5-NSA	2.36	2.45	97.77 ± 1.90	96.40 ± 2.41	99.64 ± 1.29

^1^ Analyte: mesalazine (MSZ), folic acid (FA), 4-aminosalicylic acid (4-ASA), 4-aminophenol (4-AP), 2,5-dihydroxybenzoic acid (2,5-DHB), 3-aminobenzoic acid (3-AB), 3-aminophenol (3-AP), salicylic acid (SA), 2-chloro-5-nitrobenzoic acid (2Cl5-NBA), 2-aminophenol (2-AP) and 5-nitrosalicylic acid (5-NSA).

**Table 3:** Forced degradation study results.

Degradation Condition	*t*/*h*	Standards		Standard Blend		Tablet Blend	
		% MSZ	% FA	% MSZ	% FA	% MSZ	% FA
0.1 M NaOH	1	9.4	2.1	11.6	1.4	10.4	1.6
0.1 M HCl	0.5	0.8	10.6	0.5	8.7	0.1	6.0
3% H_2_O_2_	1	9.1	0.7	9.8	0.7	10.3	1.0
Photo (solution)	4 (12 ^1^)	2.9	18.3	13.4	4.6	11.1	2.3
Photo (solid) ^2^	168	4.4	2.9	6.4	4.1	4.2	6.5
Thermal (solution)	18	15.4	n.d. ^3^	11.1	0.8	13.0	2.8
Thermal (solid) ^2^	168	9.0	n.d. ^3^	7.4	n.d. ^3^	8.6	n.d. ^3^

^1^ 12 h reaction time refers to the standard and tablet blends; ^2^ 168 h reaction time equals 7 days; ^3^ n.d. = no degradation observed.

**Table 4:** Isothermal stress testing results (after 4 weeks).

Sample	Appearance	Physical Change	Recovery (Mean ± % RSD) (*n* = 3)
MSZ tablets	Light grey powder	No significant visual changes	103.56 ± 0.21
FA tablets	Yellow powder		101.2 ± 1.56
MSZ (tablet blend)	Light yellow powder		101.73 ± 0.18
FA (tablet blend)			101.98 ± 0.62
MSZ standard	Light grey powder		99.50 ± 2.23
FA standard	Yellow powder		99.47 ± 0.69
MSZ (standard blend)	Light yellow powder		98.80 ± 0.79
FA (standard blend)			101.40 ± 1.91
